# Hydrothermal Synthesis of Binder-Free Metallic NiCo_2_O_4_ Nano-Needles Supported on Carbon Cloth as an Advanced Electrode for Supercapacitor Applications

**DOI:** 10.3390/ma15134499

**Published:** 2022-06-26

**Authors:** Qasim Abbas, Sajid Hussain Siyal, Abdul Mateen, Najam Ul Hassan, Asim Idrees, Zia Ur Rehman, ElSayed M. Tag El Din, Majed A. Bajaber, Muhammad Sufyan Javed

**Affiliations:** 1 Department of Intelligent Manufacturing, Yibin University, Yibin 644000, China; 2 Metallurgy & Materials Engineering Department, Dawood University of Engineering and Technology, Karachi 74800, Pakistan; sajid.hussain@duet.edu.pk; 3 Beijing Key Laboratory of Energy Conversion and Storage Materials, Department of Physics, Beijing Normal University, Beijing 100084, China; abdulmateen@mail.bnu.edu.cn; 4 Department of Physics, Division of Science & Technology, University of Education, Lahore 54000, Pakistan; najamphys@gmail.com; 5 Department of Applied Sciences, National Textile University, Faisalabad 37610, Pakistan; asim.idrees44@gmail.com; 6 Department of Chemistry, Hazara University, Mansehra 21120, Pakistan; ziamwt1@gmail.com; 7 Electrical Engineering Department, Faculty of Engineering & Technology, Future University in Egypt, New Cairo 11835, Egypt; elsayed.tageldin@fue.edu.eg; 8 Chemistry Department, Faculty of Science, King Khalid University, P.O. Box 9004, Abha 61413, Saudi Arabia; mb@kku.edu.sa; 9 School of Physical Science and Technology, Lanzhou University, Lanzhou 730000, China

**Keywords:** nickel–cobalt oxide, carbon cloth, nano-needle arrays, supercapacitor

## Abstract

It is of great significance to design electrochemical energy conversion and storage materials with excellent performance to fulfill the growing energy demand. Bimetallic cobalt/nickel-based electrode materials exhibit excellent electrical conductivity compared to mono oxides. However, their potential as electrode materials for high-performance supercapacitors (SCs) is limited because of their poor cycling stability and high-capacity fading. This work demonstrates the synthesis of binder-free bimetallic NiCo_2_O_4_ nano-needles supported on CC (NCO@CC) via a facile and scalable hydrothermal process. Excellent electrical conductivity and interconnected nanostructure of NCO@CC nano-needles provide the fast transfer of electrons with numerous channels for ion diffusion. Owing to such features, the binder-free NCO@CC electrode for SC discloses excellent specific capacitance (1476 Fg^−1^ at 1.5 Ag^−1^) with 94.25% capacitance retention even after 5000 cycles. From these outstanding electrochemical performances, it can be inferred that NCO@CC nano-needle array-structured electrodes may be potential candidates for SC applications.

## 1. Introduction

To fulfill the growing ultimatum of portable devices that rely on energy storage devices to function, researchers have paid close attention to energy storage to develop sustainable ways of reducing dependence on fossil fuels [1]. To this end, a set of different storage materials and devices, such as supercapacitors (SCs) and rechargeable batteries, have also been developed for various important applications [2]. Compared to batteries, SCs have many advantages: low internal resistance, long life span, high specific power, lightweight, as well as flexibility. Electrode materials have become the core components of SCs and can be generally categorized into three main types: carbonious materials, metal oxides, and conducting polymers [3]. Carbonious materials, which are inexpensive and have good electrical conductivity, excellent specific surface area (~3000 m^2^g^−1^), as well as chemical durability, are ideal for electrochemical double-layer capacitors. However, they usually have low specific capacitances (below 400 Fg^−1^) [4].

In contrast, generally employed pseudocapacitive materials are conducting polymers and transition metal oxides [5,6]. Although conducting polymers have excellent specific capacitance (below 530 Fg^−1^) and higher intrinsic conductivity, they suffer from an extremely poor life-cycle due to their significant expansion and contraction during charge–discharge [7]. Compared to the two types mentioned above, transition metal oxides usually have diverse oxidation states, which is desirable for rapid redox reactions, thus resulting in excellent specific capacitances [8]. Owing to these transition metal oxides’ inherently low electronic conductivity (band gaps 3 to 4 eV), their exceptional electrochemical performance cannot be guaranteed as long as they are integrated with other well-conducting materials, especially at ultra-high rates [9].

Among other substrates, such as nickel foam [10], carbon cloth (CC) has been feasibly employed as a self-sustaining substrate material for electrodes and reduced the ion/electron diffusion paths. CC possesses a broader application potential compared to the widely used Ni foam and Fe nanostructures [11]. However, it is essential to combine high-capacity electrode materials and explore their advantages [12]. Pseudocapacitive materials are highly used electrodes, such as oxides, hydroxides, and sulfides of transition metals [13,14], yet their applications are limited, owing to their poor electrical conductivity as well as cycling stability [15]. To further enhance electrochemical performance, research has progressed to constructing bimetallic nanostructures. Particularly, the strategy of directly growing various nanostructured pseudocapacitive materials on conductive substrates is considered to have large possibilities [16]. Compared to single transition metal-based electrode materials, multi-component composite transition metal oxide-based electrode materials exhibit superior electronegativity, conductivity, and cycling stability [17].

Among various pseudocapacitive electrodes, nickel-cobaltite (NiCo_2_O_4_) is examined as an encouraging electrode because of its greater electrochemical activity and theoretical capacitance than individual constituent nickel or cobalt oxide [18]. The electrochemical advantage of NiCo_2_O_4_ stems from the redox couple contributed by NiO and CoO that makes multi-electron redox reactions during charge–discharge [19,20]. It has been reported that the capacitance of NiCo_2_O_4_-based electrodes exhibits 58% and 130% more than the electrodes based on NiO and CoO, respectively [21]. NiCo_2_O_4_ has garnered much because of its easy synthesis, low toxicity, and excellent electrochemical response [20]. Considerable attempts have been made to enhance the electrochemical performance of NiCo_2_O_4_ for SCs by tuning the morphologies of nanosheets [22], nanowires [23], nanotubes [24], and microspheres [25]. Nonetheless, the structural collapse and volume expansion of NiCo_2_O_4_-based materials are inevitable in charge–discharge. Still, they suffer from unstable cycling performance as well as low power density to meet novel and high requirements for next-generation applications [26,27]. Integrating NiCo_2_O_4_ nanostructures onto substrates can overcome these problems, improving the pseudocapacitive performance. Thus, directly immobilizing NiCo_2_O_4_ nanostructures on CC with tailored content and morphology is highly desirable.

Herein, we demonstrate the synthesis of binder-free bimetallic NiCo_2_O_4_ nano-needles supported on CC (denoted as NCO@CC) via a facile and scalable hydrothermal process. The excellent electrical conductivity and interconnected nanostructures of NCO@CC transfer electrons from electroactive elements to external circuits, numerous channels for ion diffusion, and more active sites. Due to such features, the binder-free NCO@CC electrode for SC discloses an excellent specific capacitance (1476 Fg^−1^ at 1.5 Ag^−1^) with long-lasting retention of 94.25%, even after 5000 cycles. This work demonstrates that binder-free NCO@CC nano-needle array-structured electrodes may be potential candidates for SC applications.

## 2. Experimental Section

### 2.1. Initial Treatment of CC

Initially, CC of 1 × 2 cm^2^ was washed ultrasonically in acetone, ethanol as well as deionized (DI) water for 15 min and the oil and oxide layer on the CC surface was completely removed. The washed CC were then dehydrated overnight at 60 °C. To make CC more hydrophilic, NCO@CC was oxidized in KMnO_4_ (1 mg/mL) and H_2_SO_4_ (5%) solution at 50 °C for 1 h and then transferred to a solution of H_2_O_2_ and HCl to remove residual manganese oxides. Finally, a hydrophilic electrode was thoroughly washed with ethanol and DI water and dried in a vacuum oven at 110 °C.

### 2.2. Typical Fabrication Mechanism

The NCO@CC precursor was prepared via the facile and straightforward hydrothermal strategy. A total of 0.36 g Ni(NO_3_)_2_·6H_2_O, 0.72 g Co(NO_3_)_2_·6H_2_O, 0.9 g NH_4_F and 0.9 g urea was dissolved and stirred in 50 mL DI water at room temperature (RT) for 1 h until the color of solution changed to uniform pink. Then, the pretreated CC and mixed solution were transferred to a 100 mL Teflon container, which was sealed in a stainless-steel autoclave, and then reacted hydrothermally in an oven at 135 °C for 10 h. After that, the NCO@CC was ultrasonically washed many times, first with ethanol and then DI water, thereby removing the residual NiCo_2_O_4_ on CC, and then dried overnight in an oven at 60 °C. Finally, NCO@CC was obtained and ready for further analysis and electrochemical investigations as a positive electrode for SC.

### 2.3. Assembly of the Positive Electrode for SC

The as-prepared NCO@CC was used as a working electrode (active area ~1 × 1 cm^2^). The mass loading of NCO on the CC was 1.2 mg cm^−2^. Ag/AgCl was employed as a reference electrode, while platinum wire as a counter electrode in 6 M KOH-based aqueous electrolyte.

### 2.4. Physical Analysis and Characterization

Surface microarchitecture of the sample was analyzed via field emission scanning electron microscopy (FESEM, HITACHI SU8220) and transmission electron microscopy (TEM JEM-2100F, JEOL). The crystal structure was investigated using an X-ray diffractometer (X’Pert Pro PANalytical) with monochromated Cu-Kα radiations (λ = 0.15406 nm). The Brunauer–Emmett–Teller (BET) and Barret–Joyner–Halenda (BJH) analysis curves were obtained to investigate the surface area and pore size with Micromeritics ASAP2460 analyzer. To investigate the chemical bonds, the Raman spectrum (HJY Lab RAM Aramics 70, France) was used.

### 2.5. Electrochemical Characterization

Fabricated samples were electrochemically inspected via cyclic voltammetry (CV), galvanostatic charge–discharge (GCD) investigation, and electrochemical impedance spectroscopy (EIS) by an electrochemical work station (CHI 660E, Wuhan, China) using a standard three-electrode system. The CV were carried out in a voltage window range of 0 to 0.6 V; GCD investigations were carried out at various current density ranges from 1.5 to 50 Ag^−1^. EIS at different frequencies from 0.001 to 100 kHz was studied.

The specific capacitance (C_sp_ (Fg^−1^)) was computed by the equation given below [28]:

(1) $$C_{\mathrm{sp}}=\frac{1}{mv\left(V_{f}-V_{i}\right)}\;\overset{V_{f}}{\underset{V_{i}}{\int }}I\;dV$$

where *m*(g), *v*(mVs^−1^), *I* *dV*, and Δ*V*(*V_f_* − *V_i_*) are active material’s mass, scan rate, area under discharge curve, and applied voltage window, respectively.

The charge storage efficiency (η) was computed using the following equation:

$$\eta \;\left(\%\right)=\frac{\int_{V_{i}}^{V_{f}}I_{d}\;dV}{\int_{V_{i}}^{V_{f}}I_{c}\;dV}\;\times 100$$

where the terms *I_d_ dV* and *I_c_ dV* represent the area under charging curves and discharging curve, respectively.

## 3. Results and Discussion

**Figure 1:**
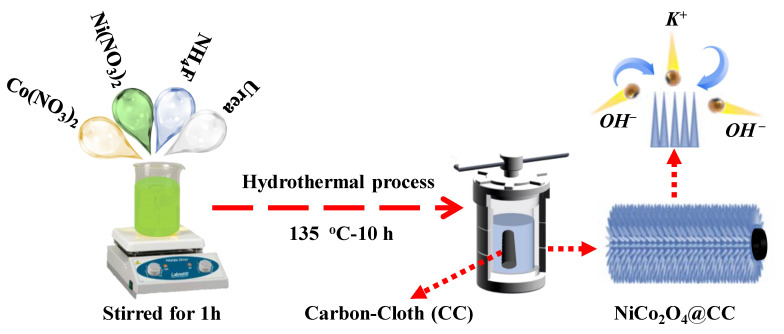
Schematic diagram of the preparation method of NCO@CC nano-needles.

The synthesis strategy of NiCo_2_O_4_ on the conductive surface of CC is schematically illustrated in Figure 1, where the NiCo_2_O_4_ has directly grown at CC (denoted as NCO@CC) via a simple and scalable hydrothermal method at RT. After that, NCO@CC was annealed at 135 °C for 10 h with a ramping rate of 2 °C min^−1^ to synthesize mechanically stable NCO@CC nano-needles. The upper part (Figure 1) shows a magnified view of the electrolyte ion flow in NCO@CC nano-needles in a KOH-based aqueous solution.

**Figure 2:**
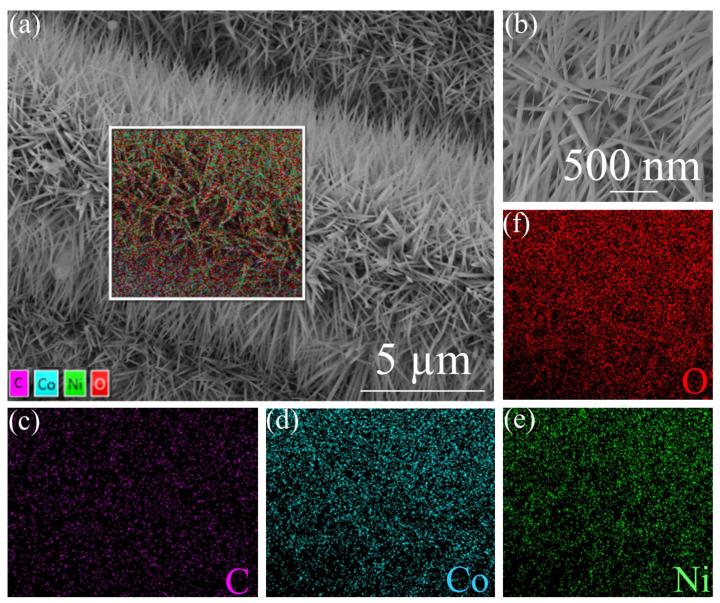
SEM image of NCO@CC nano-needles: (**a**) low magnification, (**b**) high magnification, (**c**–**f**) corresponding EDS elemental mapping of NCO@CC nano-needles.

SEM was used for the structural and morphological investigations of NCO@CC. Figure 2a shows the low-magnification SEM image of NCO@CC with the existence of densely ordered nano-needles of NCO@CC. A similar morphology can be seen in the high-magnification SEM image, where NiCo_2_O_4_ nano-needles are homogenously loaded at the CC surface (Figure 2b), suggesting the successful grafting and excellent adhesion of NiCo_2_O_4_ nano-needles on CC.

Such morphological structures of NCO@CC nano-needles may facilitate and enhance the electron and ion transmission in the electrolyte. Further, energy-dispersive X-ray spectroscopy (EDS) shows similar distribution and homogeneous dispersion of C, Co, Ni, and O elements in NCO@CC nano-needles. The CC is composed of carbon fiber with good electrical conductivity and flexibility. NH_4_F and urea, as alkali sources limit the nucleation as well as crystallization of NiCo_2_O_4_, effectively controlling its size [29,30]. Thus, the amount and type of alkali sources play an essential role in regulating the NiCo_2_O_4_ morphology.

**Figure 3:**
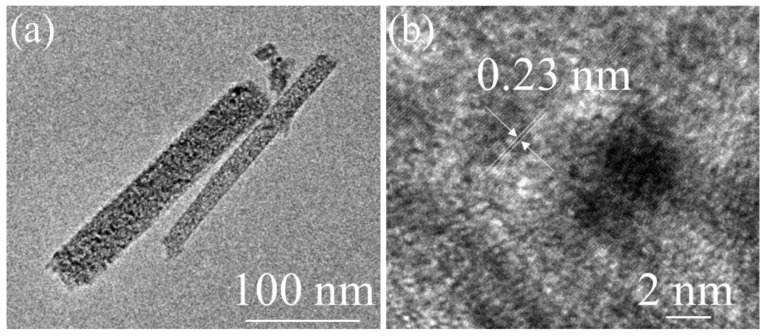
(**a**) TEM image of NCO@CC nano-needles, (**b**) HRTEM image with lattice fringes.

To further investigate the detailed morphology and microarchitectures of NCO@CC nano-needles, high-resolution TEM (HRTEM) was performed. Plentiful mesopores were uniformly distributed over the entire NCO@CC nano-needles, indicating that the as-fabricated NiCo_2_O_4_ nano-needles have mesoporous features upon hydrothermal treatment, as shown in Figure 3a. Obviously, the lattice distance of 0.23 nm corresponds to (220) planes of the spinel NiCo_2_O_4_ phase, as shown in Figure 3b. Therefore, the porous features and superior morphology of NCO@CC nano-needles, as a positive electrode material, are beneficial for improving the stability and electrochemical performance.

**Figure 4:**
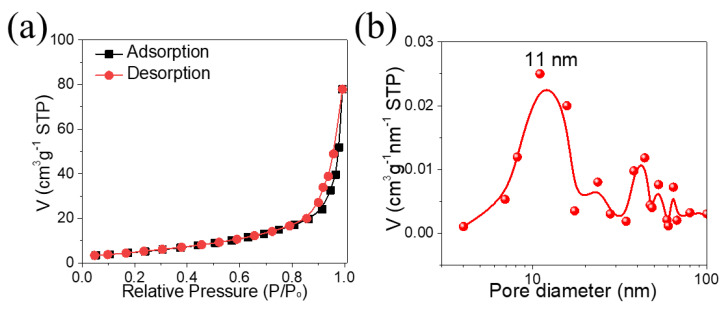
(**a**) N_2_ adsorption–desorption isotherms of NCO@CC nano-needles, (**b**) the corresponding pore size distribution.

The nitrogen (N_2_) adsorption–desorption analysis was carried out to find the surface area along with pore size distribution of NCO@CC nano-needles. Figure 4a shows the N_2_ isotherm of NCO@CC nano-needles, a Langmuir type-IV sorption isotherm, and the H4 hysteresis loop as stated in IUPAC classification [31]. The present hysteresis loop indicates that NCO@CC nano-needles have a distinct mesoporous architecture. The BET-specific surface area of NCO@CC nano-needles was calculated to be 37 m^2^g^−1^. The BJH pore size distribution further confirms that NCO@CC nano-needles were dominated by mesopores (Figure 4b), and the results were consistent with the TEM analysis. The mean pore size was equal to about 11 nm, indicating the presence of greater pore size. These mesoporous architectures, along with a greater surface area, can offer good electrochemical performance as well as more electroactive sites, favorable to electrolyte penetration and fast ion and electron transport [32].

**Figure 5:**
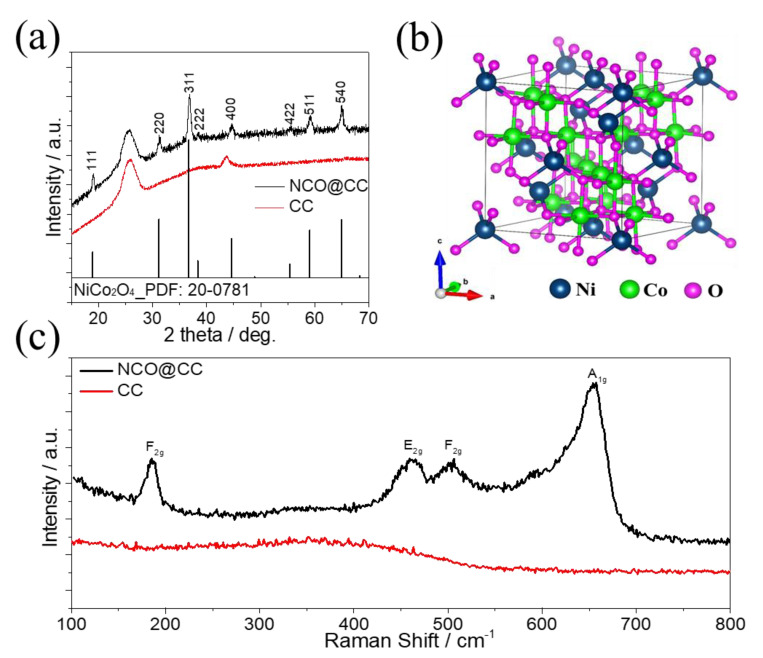
(**a**) XRD patterns of NCO@CC nano-needles, (**b**) crystal structure of spinel NiCo_2_O_4_, (**c**) Raman spectrum of NCO@CC nano-needles.

The XRD patterns of the NCO@CC nano-needles are illustrated in Figure 5a. All characteristic peaks are associated with the XRD pattern of spinel NiCo_2_O_4_ (PDF card number 20-0781) [33], while a strong XRD peak position (25.5°) is associated with CC (002). The characteristic peaks located at 19.1°, 31.3°, 36.8°, 38.9°, 44.7°, 55.1°, 58.1°, and 64.8° are associated with (111), (220), (311), (222), (400), (422), (511) and (440) planes of NiCo_2_O_4_, respectively. It makes the NiCo_2_O_4_ nano-needles fabricated on the CC surface more homogeneously anchored. In addition, XRD data confirm the formation of the NiCo_2_O_4_ phase without any impurities, such as NiO, CoO, and Co(OH). Figure 5b exhibits the spinel crystal structure NiCo_2_O_4_ (space group Fd3m), where Co atoms are embedded at octahedral and tetrahedral sites, whereas Ni atoms are distributed over octahedral sites. The Raman spectrum of NCO@CC nano-needles (Figure 5c) exhibits vibrational peaks at 187, 462, 506, and 658 cm^−1^ associated with F_2g_, E_g_, F_2g_, and A_1g_ systems of NiCo_2_O_4_, respectively. The Raman spectrum of NCO@CC nano-needles is in good agreement with already reported values [34,35]. It only demonstrates vibrations of Ni-O and Co-O, indicating that the nickel–cobalt hydroxycarbonate precursor was decomposed into NiCo_2_O_4_.

**Figure 6:**
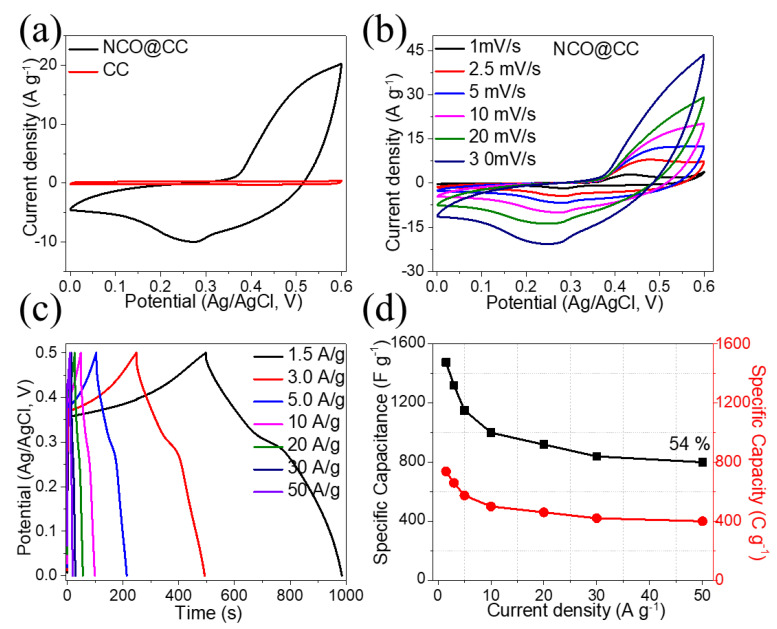
(**a**) CV profiles of NCO@CC as well as CC electrode, (**b**) CVs of NCO@CC electrode, (**c**) GCD profiles, (**d**) specific capacitance and capacity as a function of current density.

Electrochemical measurements of the NCO@CC electrode were performed via a three-electrode mode in a 6 M KOH aqueous electrolyte. Figure 6a displays the CV profiles of NCO@CC and CC in the potential window range from 0 to 0.6 V. Clearly, the current rate as well as the neighboring area of pure CC electrode are much lower compared to the NCO@CC, indicating that the capacitance of CC is almost small. The CVs of the NCO@CC electrode show the prominent redox peaks derived from the Faradaic reaction of Ni and Co ions mediated by OH^−^ ions in an electrolyte, indicating that the Faradaic redox reaction offers its capacitance. In addition, CVs show a larger area, which means they exhibit higher specific capacitance and excellent electrochemical performance due to the more rational Co/Ni ratio enhancing the synergistic effect between Ni and Co ions. Figure 6b shows typical CV profiles of NCO@CC electrode with different scan rates from 1 to 30 mV s^−1^ in the voltage window range from 0 to 0.6 V. The shape of CVs is the same as the previous NiCo_2_O_4_ reports in KOH solution [36]. The pseudocapacitive response of the NCO@CC electrode can be confirmed by two redox peaks that can be perceived in all CV profiles and cannot be seen in electric double-layer capacitance. Further, at 1 mVs^−1^, these oxidation and reduction peaks were seen at 0.47 V and 0.42 V, respectively. By increasing the scan rate (from 1 to 30 mV s^−1^), oxidation peaks were shifted to higher potentials while the reduction peaks shifted to lower potentials [37]. The redox peaks can be associated with the M–O/M–O–O–OH, where M shows Ni and Co ion reactions related to OH^−^ anions. This indicates that reaction kinetics are reversible due to the polarization and ohmic resistance of active material given during the redox process. The GCD profiles of the NCO@CC electrode in the voltage window range from 0 to 0.5 V and are shown in Figure 6c. The robust non-linear GCD profiles of the NCO@CC electrode are preserved, demonstrating the representative response of the Co^2+^/Co^3+^ and Ni^2+^/Ni^3+^ redox couples at current density ranges of 1.5–50 Ag^−1^ [38]. The voltage (IR) drop for all current densities from 1.5 to 20 Ag^−1^ is lower than 100 mV, and it is 150 mV for 50 Ag^−1^. Highly symmetrical and prolonged discharge times at high current densities indicate good Coulombic efficiency as well as good charge storage properties. The specific capacitance (Fg^−1^) and specific capacity (Cg^−1^) of the NCO@CC electrode obtained from the GCD profiles at current rates from 1.5 to 50 Ag^−1^ are illustrated in Figure 6d. Specific capacitance of the NCO@CC electrode was 1476 Fg^−1^ (738 Cg^−1^) at 1.5 Ag^−1^. As the current rate enhanced up to 50 Ag^−1^, the specific capacitance of the NCO@CC was still as high as 800 Fg^−1^(400 Cg^−1^), which is 54% of the retention capacitance. The charge storage efficiency (η) of the electrode was >90% at all current densities, specifically 92% at 1.5 Ag^−1^ and 93.2% at 50 Ag^−1^. These excellent results are due to the unique architectures of interlinked porous NCO@CC nano-needles.

**Figure 7:**
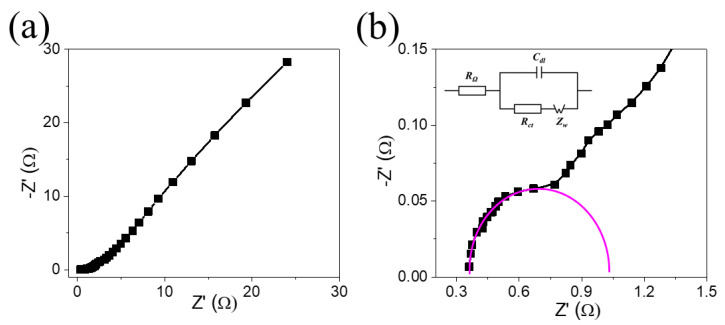
(**a**) Nyquist plot NCO@CC, (**b**) an extended view of the high-frequency plot with an inset image is an equivalent circuit diagram.

For the electrochemical response of NCO@CC electrodes, an EIS investigation was carried out to examine the corresponding mechanism on the electrode surface. The Nyquist plot shows a semicircle of the high-frequency section with a straight line in the low-frequency section, as shown in Figure 7a. The intersection between the curve and the real axis represents equivalent series resistance (R_s_), such as the inherent resistance of the electrode, the electrolyte ionic resistance, and the contact resistance of the material. In the high-frequency region, the diameter of the semicircle is associated with the electrode’s charge transfer resistance (R_ct_). In the low-frequency section, the straight line shows the Warburg impedance Z_w_ of diffusion of electrolyte, the slope of which is related to the ionic diffusion of the electrolyte to the surface of the electrode [39]. Here, the NCO@CC electrode has R_s_ value (0.36 Ω) and R_ct_ (0.74 Ω), indicating high electrical conductivity and low internal resistance. Furthermore, the NCO@CC electrode showed the most significant straight-line slope in the low-frequency section.

The behavior of the NCO@CC electrode in the high-frequency part is illustrated in Figure 7b, exhibiting an expanded view of the NCO@CC electrode. In the high-frequency section, the plot clearly shows a semicircle with a straight line in the low-frequency part. The equivalent circuit is used to find the R_ct_ of the NCO@CC (Figure 7b, inset). These experimental results demonstrate that the NCO@CC nano-needle electrode has outstanding electrochemical performance, which helps to provide the smallest internal resistance as well as remarkable electrolyte–ion diffusion ability.

**Figure 8:**
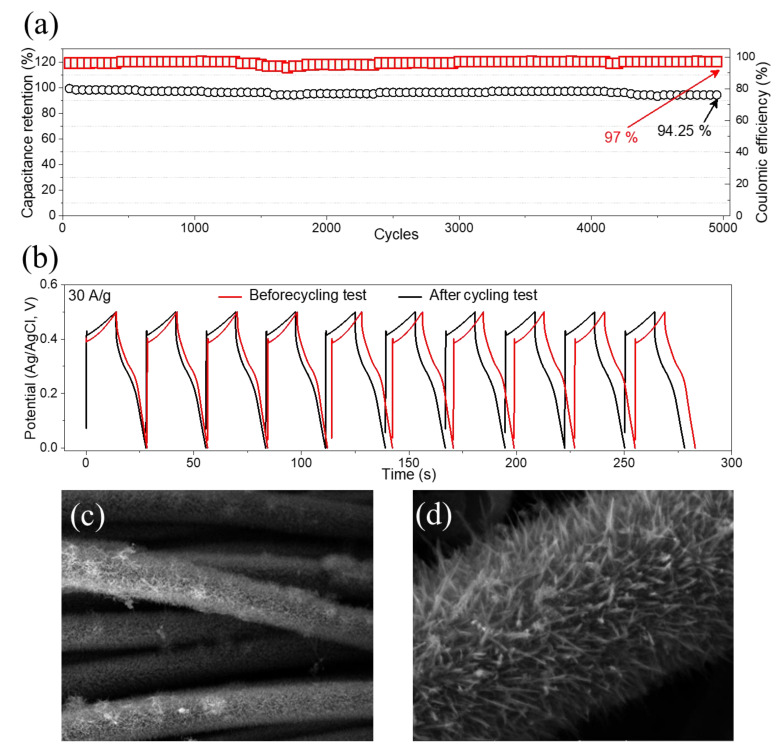
(**a**) Cycling performance of NCO@CC electrode, (**b**) first and last 10 cycles, (**c**,**d**) SEM images after cycling stability tests.

The cycling performance investigation is an essential requirement for SCs. Thus, the cycle stability investigations of the NCO@CC were performed using GCD cycling (Figure 8a). This shows that the capacitance of the NCO@CC electrode decreased slightly after 1500 cycles and became stable until 2500 cycles. Afterward, a slight improvement in capacitance can be seen, which was further improved after 3200 cycles, attributed to the activation of the material. The NCO@CC electrode exhibited 94.25% retention capacitance over 5000 GCD cycles. This capacitance retention indicates the ultra-high stability and superior energy storage performance during GCD cycles of the NCO@CC. Furthermore, the stability of the NCO@CC electrode was investigated through 5000 GCD cycles at 30 Ag^−1^ before and after the cycling test, in which the GCD curves were almost the same (Figure 8b). This shows that the fabricated NCO@CC electrode exhibited excellent stability after 5000 cycles. SEM analysis was conducted after the cycling stability test, and the corresponding low- and high-resolution images are shown in Figure 8c,d, respectively. The SEM images show the good connection of NCO with CC substrate and retained almost the same morphology without any severe deformation or degradation, indicating the excellent stability of the electrode in the KOH electrolyte.

The NCO@CC electrode presents excellent electrochemical performance because of a number of reasons. (1) The NCO@CC is highly electrical conducting; (2) the synergistic effect of Ni with Co elements is favorable for the increase in adsorption energy of the electrolyte ions (OH^−^) [40]; (3) the porous nano-needle arrays of the NCO@CC electrode possess greater surface area, as well as good aspect ratio that can offer rich active sites for excellent capacity; (4) sustainable and stable architecture configurations by NiCo2O4 on CC are favorable for promoting rapid electron and ion transport.

## 4. Conclusions

To conclude, we have demonstrated interconnected porous nano-needle arrays such as bimetallic NiCo_2_O_4_ supported on carbon cloths (NCO@CC) by adopting a simple and scalable hydrothermal synthesis. The NCO@CC electrode demonstrates good electrochemical performance in a three-electrode mode, and a gravimetric capacitance of 1476 Fg^−1^ at a current rate of 1.5 Ag^−1^ was obtained. Furthermore, NCO@CC attains 94.25% of retention capacitance over 5000 GCD cycles. Assembly of nano-needle structures ensures complete electrochemical utilization of the NCO@CC electrode in charge storage, as it facilitates fast electron and ion transport. The unique structure of the electrode largely reduced the ion diffusion resistance as well as charge transfer, with excellent rate capability, specific capacitance value, and cycling performance. These findings prove that NCO@CC nano-needle-like electrode is promising with a tunable electrochemical performance, which aspired its commercialization for the development of advanced SCs.

## Data Availability

All data is provided in this paper.
